# Comprehensive Analysis of E478 Single-Component Epoxy Resin and Tungsten-E478 Interface for Metallic-Polymer Composite Electron Source Applications

**DOI:** 10.1021/acsomega.4c04052

**Published:** 2024-06-29

**Authors:** Mohammad M. Allaham

**Affiliations:** Central European Institute of Technology, Brno University of Technology, Purkyňova 123, Brno 612 00, the Czech Republic; Institute of Scientific Instruments of Czech Academy of Sciences, Královopolská 147, Brno 612 64, the Czech Republic; Department of Physics, Faculty of Electrical Engineering and Communication, Brno University of Technology, Brno 61600, the Czech Republic

## Abstract

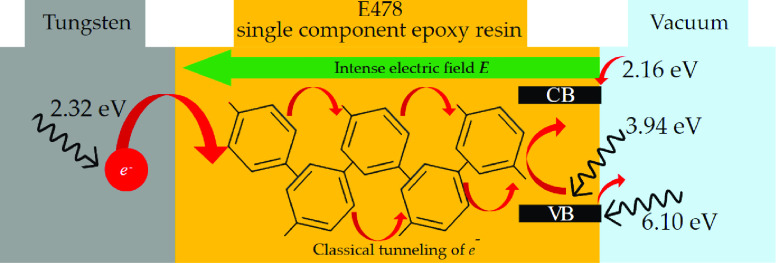

This study provides comprehensive elemental, optical, and energy gap characteristics of the E478 single-component epoxy resin. This type of epoxy resin has imperative applications in medium voltage insulation and cold field emission of electrons. X-ray photoelectron spectroscopy (XPS), Raman spectroscopy, and hydrogen nuclear magnetic resonance (1H-NMR) were used to study the elemental and structural analyses, ultraviolet photoelectron spectroscopy (UPS) was used to obtain the local work function and the ionization potential energies, and ultraviolet/visible light spectroscopy (UV/VIS) was used to report the optical and energy gap characteristics of the epoxy resin being studied. Moreover, the UPS and UV/VIS analyses were merged to obtain the electron affinity of the E478 epoxy resin and to study the epoxy’s energy band diagram and the tungsten-epoxy interface band structure. The results showed that the E478 epoxy resin is considered an n-type semiconductor of energy gap ∼3.94 eV, local work function ∼3.42 eV, ionization potential ∼6.10 eV, electron affinity ∼2.16 eV, and tungsten-epoxy Schottky contact barrier height ∼2.50 eV.

## Introduction

Epoxy resins are widely used in various applications thanks to their advantageous properties, such as their chemical resistance and mechanical properties.^1−3^ Among these applications, epoxy resins have been applied as coating materials in studying the cold-field emission of electrons. The coating layer acts like a tunneling layer that separates the metal-vacuum interface. Moreover, it protects the cathode surface from unwanted environmental factors affecting the emission process, such as ion bombardments on its surface.^4,5^

**Figure 1:**
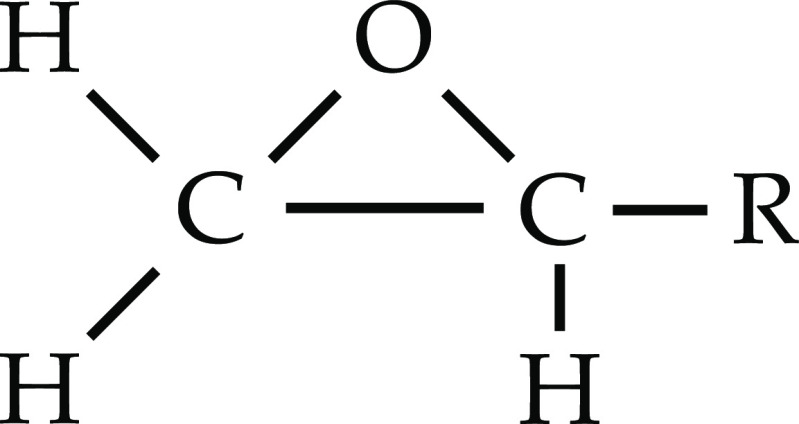
Oxirane chemical structure (R is an organic functional group).

In general, epoxide functional groups (oxiranes) are three-membered rings of two carbon atoms and an oxygen atom (C–O– C). The ring is synthesized by the oxidation process of unsaturated functional groups such as alkenes (e.g., oxidation of ethylene or propylene). The general form of an epoxide group structure is presented in Figure 1.^6^

Epoxy resins are highly viscous substances containing low molecular weight prepolymers. The included prepolymers contain reactive groups with more than one oxirane group. Epoxy monomers are functional groups containing one oxirane group that is used to build epoxy resins. In the global epoxy resin manufacturing market, the most used epoxy resin as the base component is the bisphenol A diglycidyl ether (DGEBA), which presents 50–60% the base of single-component epoxy resins and 90% of the two-components epoxy resins.^7,8^

In chemistry, the curing process converts a lower molar-mass polymer to another polymer of higher molar-mass.^9^ The curing process of epoxy resins describes the reaction between the resin and its curing agent when it is induced by an equivalent activation method, such as heat. During this process, the high-viscosity liquid is converted to a thermoset of a highly cross-linked network. Thus, the term ”epoxy resin” describes a class of thermosetting polymers.^10,11^

**Figure 2:**
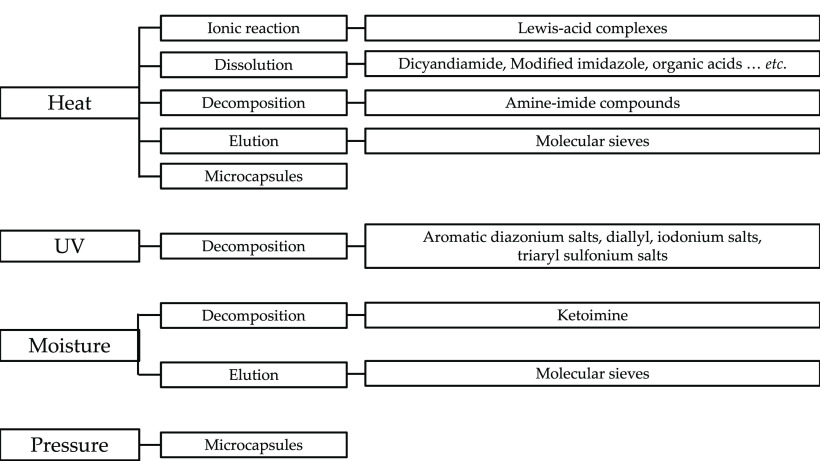
Diagram presenting the single-component epoxy activation methods, phenomena, and related curing agents.

Epoxy adhesives have two forms, regular two-component epoxy adhesives that include separated epoxy resin and curing agent (hardener) and one-component (or one-part, single-component) epoxy resins that include mixed resin and latent curing agent. Various activation methods can be used to cure one-component epoxy resins. Therefore, choosing a curing agent depends on the selected activation method, activation phenomena, and state of the curing agent (whether it is latent or not). Check Figure 2 for more information regarding the curing methods.^7,12,13^

Thixotropy is an important property of one-component epoxy resin since mechanical stirring reduces its viscosity. However, after removal of the mechanical stress, the resin will return to its initial state. Thixotropic epoxy resins are easier to apply on surfaces because they spread easily, allowing higher coverage and deeper impregnation of the resin inside the target material.^14,15^

Cold field electron (CFE) emission is a quantum mechanical tunneling phenomenon. In this phenomenon, an intense electrostatic field is applied between two electrodes separated by a vacuum gap. The cathode is prepared as a micro/nano single tip field emitter (STFE) or an array of STFEs known as large area field emitters (LAFE). The applied electrostatic field reduces the potential energy barrier at the surface of the cathode apex, which allows cold electrons (electrons with low energy at the Fermi level) to quantum mechanically tunnel through to form an electron beam.^16,17^

As one of the single-component epoxy resin applications, Mousa *et al*. applied thin layers of different epoxy resins to tungsten STFEs. The coating layer acted as an additional quantum barrier through which the emitted electrons needed to tunnel through. The results showed unique switch-on behavior (sudden emission currents of intense current densities) of the emission process that depends on the thickness of the applied layer, and the resulting electron beam was more concentrated, higher in brightness, operating at lower threshold voltages, having different energy distributions and different current–voltage characteristics. However, the mechanism behind the changes in the typical characteristics of a CFE process is still not fully understood.^18−23^

In this article, we study the optical and electrical structure characteristics of the Elantas epoxylite E478 single-component epoxy resin (E478 for simplicity). This is important to provide a new possible theory for the emission process from composite metal–polymer electron sources. X-ray photoelectron spectroscopy (XPS) was used to study the elemental analysis of the E478 epoxy resin, and the chemical bonds were checked by Raman spectroscopy and hydrogen nuclear magnetic resonance (1H-NMR). However, the chemical formula of this epoxy resin is not publicly available. Thus, the results focused on basic elemental analysis. The ionization potential energy (ψ), the local work function (ϕ), and the edge of the highest occupied energy level of the valence band (*E*_HOMO_) were determined from the ultraviolet photoelectron spectroscopy (UPS). The optical analysis and energy gap measurements were obtained using ultraviolet/visible light spectroscopy (UV/VIS). Moreover, high purity (99.99%) tungsten sheets were coated with a thin film of the E478 epoxy resin to study the tungsten-E478 interface. Finally, a new model describing the CFE and switch-on phenomena from tungsten-E478 composite cathodes is introduced.

## Materials and Methodology

### Materials

Elantas PDG epoxylite E-478 single-component thixotropic epoxy VPI resin (E478) (produced by Elantas Europe) is an industrial one-component epoxy adhesive used for electrical insulation applications in the medium voltage range (<7 kV). Moreover, thanks to its high chemical resistance, it is used in coating applications for protecting surfaces in corrosive environments. The thixotropic property of the E478 epoxy is essential for film building and resin retention on the substrate surface. Furthermore, vacuum impregnation is an even more important property because it helps deeper resin penetration within the surface topography.

According to the safety data sheet (SDS), E478 has Poly(Bisphenol A-*co*-epichlorohydrin) as the prepolymer, Neopentyl glycol diglycidyl ether as the epoxy diluent and the source of the oxirane functional group, Polyglycol as a thermosetting enhancement factor, and Trichloro(N,N-dimethyloctylamine)boron as a catalyst to enhance the curing process. The E478 epoxy resin can be cured in different temperature–time curing conditions, such as 149 °C for 6 h or 169 °C for 4 h. The dielectric strength of this type of epoxy is 0.15 V/nm as provided in the product datasheet, where this value was measured following the international designation: D149. The relative permittivity of this epoxy has a value of 3.5 at 1 kHz-100 °C.

**Figure 3:**
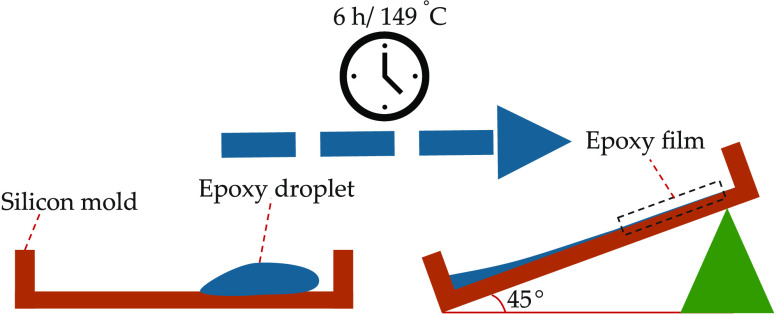
Schematic diagram explaining the E478 film preparation method.

The E478 films were prepared by setting the curing temperature at 149 °C for 6 h. The films had average thicknesses of ∼120, 180, 130, and 170 μm. The films were left inside the furnace for 2 h to cool down and prevent surface hairline cracks. Because of the thixotropic nature of the E478 epoxy resin, the films were prepared in silicon molds that were tilted by 45°, which is necessary to prevent shrinking the epoxy resin during the curing process, as seen in Figure 3.

To study the tungsten-E478 interface, high-purity (99.99%) tungsten foil (produced by Goodfellow, Hamburg, Germany) with a thickness of 0.2 mm was used as the substrate for the epoxy films. The coating process was the same as that for the film preparation.

### Methodology

#### Elemental Analysis

**Figure 4:**
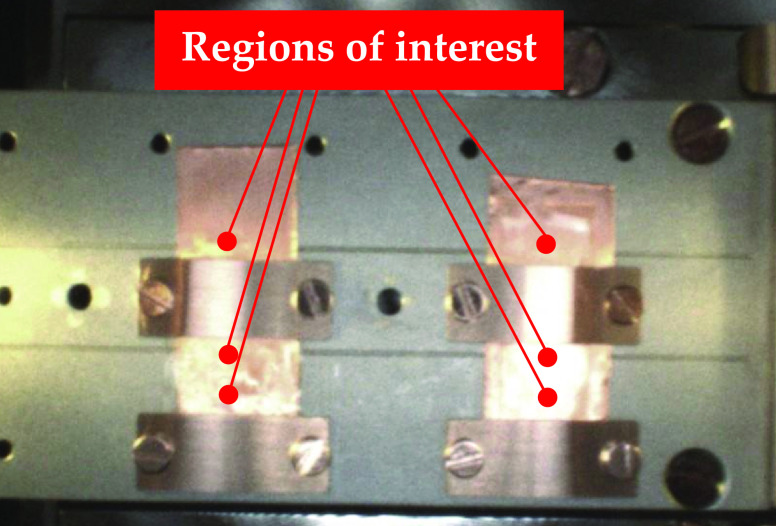
Region of interest for XPS and UPS analyses. The scanned areas were chosen to have the best surface electrical conductivity and no copper contribution in the photoemission of electrons.

The AXIS Supra X-ray photoelectron spectrometer was used to obtain the XPS spectra. To ensure the electrical conductivity for the scanned area for XPS and UPS, conductive copper bars were used to connect the scanned surface to the stage as presented in Figure 4. Moreover, the XPS spectra of the scanned region showed no contribution from the copper bars in the photoemission process.

In this study, the monochromatic source is the Al anode Kα with an energy of 1486.6 eV. The spectra were achieved using an emission current of 20 mA and collimation of a 110 um aperture. The resolution for the wide spectra was set to 80 eV, and five sweeps were performed for the wide scan. The charge neutralizer was enabled with 0.45 A of filament current, 1.05 V of filament bias, and 4.6 V of charge balance. The high-resolution spectrum of C_1*s*_ was obtained by setting the resolution of the scan to 20 eV, and 10 sweeps were performed to smooth the obtained spectrum.

Moreover, a WITec confocal Raman imaging system alpha300 R (Anton paar co., Bratislava, Slovakia) was used to study the Raman spectra from the prepared films. A green laser with 532 and 578 nm excitation and central wavelengths was used. The scanning power was 20 W, and 5 s integration time accumulations were accomplished. Furthermore, the Spinsolve 60 benchtop NMR spectrometer (Magritek, Aachen, Germany) was used to study the 1H-NMR for the uncured epoxy resin.

#### UPS

The UPS results were obtained from the same instrumentation as the XPS, where 3 sweeps were performed at 100 ms dwell time for each scan. The UPS analysis was performed with HeI UV source of photon energy *hν* = 21.22 ± 0.12 eV.

From the UPS spectra, the secondary electron cutoff energy *E*_SECO_ for the emitted electrons was measured along with the *E*_HOMO_ and the edge of the Fermi level *E*_F_. The film surface was etched by a 10 keV Ar500+ beam to ensure a clean measuring spot without contamination. Thus, an average value for ϕ and ψ was obtained using the following equations:^23^
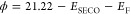
1
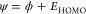
2

#### UV/VIS

The ultraviolet–visible spectroscopy is used to obtain the transmission and reflection of electromagnetic radiation passing through the films, which are obtained in the form of transmittance percentage *T*(λ)_%_ and reflectance percentage *R*(λ)_%_ functions.

These results are then used to obtain the absorption coefficient function α(λ) . For a film of thickness *d*, α value is related to the true transmittance *T* = *T*(λ)_%_/100 and true reflectance *R* = *R*(λ)_%_/100 through the following equation:^24^
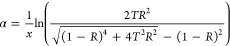
3Tauc equation is used to obtain allowed/forbidden direct and indirect optical transitions of electrons when the film is subjected to a specific radiation. Assigning *A*_0_ as the band tailing parameter (which is an energy independent constant), *E*_g*n*_ as transition-related optical energy gap and *n* as the transition type indicator, Tauc equation can then be expressed as
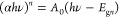
4In eq 4, the values of the exponent *n* are assigned as *n* = 2 for an allowed direct transition, *n* = 1/2 for an allowed indirect transition, *n* = 2/3 for a forbidden direct transition, and *n* = 1/3 for a forbidden indirect transition. The direct energy gap is related to opposite band edges located at the same momentum *k* value in the *k*-space, where the creation of an exciton is related to a transition without changing the momentum of the transmitted electron. The indirect energy gap is related to two adjacent band edges in the *k*-space, where the generation or recombination process is related to an electron–phonon interaction that is necessary to conserve momentum and energy during the interaction. Forbidden transitions are related to transitions from/to delocalized energy states inside the energy gap, which are related to defects in the energy band structure of the amorphous epoxy resin.

Moreover, eq 4 presents the theoretical form of the so-called Tauc plot (*α hν*)^*n*^ vs *hν*, to determine the direct and indirect energy gaps. The related energy gap is then obtained from the *X*-axis intercept of the linear part from the obtained Tauc plot. However, when the Tauc plot curve does not start from the *x*-axis, the offset correction is considered, and the energy gap value was measured at the intercept between the new offset line and the fitted line.

Epoxy resins have an amorphous structure. The disorder of their crystalline structure is related to another important parameter that measures the defects in the energy band structure and the corresponding delocalized energy states. In the ρ-space (ρ is the density of states), the delocalized energy states are presented as energy states located inside the energy gap above the valence band and below the conduction band. When the epoxy is irradiated by electromagnetic radiation, these delocalized states present defects in the energy band structure and are responsible for trapping electrons at the band edges inside the band gap.

Urbach band tail energy *E*_U_ is a parameter to study the effects of energy band defects, and it can be measured from the first Urbach empirical rule:^24^
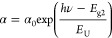
5Applying the natural logarithm to both sides of eq 5 and arranging the terms yield to obtain the following linearized form:^24^
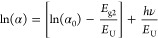
6From eq 6, the slope *S*_U_ = d(ln(α))/d(*hν*)of the linear part of the ln(α)vs *hν* plot is used to measure the band tail energy, where *E*_U_ = 1/*S*_U_. Moreover, the strength of the electron–phonon interaction *E*_*e*–*p*_ is an important parameter to describe the scattering and mobility of charge carriers in semiconducting materials, where higher values of *E*_*e*–*p*_ are related to a higher number of scattering and energy transfer events affecting the mobility of the electrons within the structure of the material. The *E*_*e*–*p*_ is measured using the second Urbach equation that connects α to the temperature *T́* of the film and the incident photons energy *hν*:^25,26^
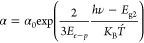
7The measurements of this study were all performed at room temperature where *T́* = 298 K and *K*_B_*T́* = 25.7 meV. Substituting these values and applying the natural logarithm to both sides yield the form:
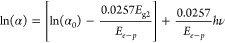
8From eq 8, the electron–phonon interaction is measured from the slope *Ś*_U_ = d(ln(α))/d(*hν*) of the linear part of ln(α) vs *hν* plot.

Moreover, comparing eq 6 to eq 8 yields the relation *E*_*e*–*p*_ = 25.96*E*_U_ at room temperature. The connection between *E*_*e*–*p*_ and *E*_U_ shows that the electron–phonon interaction is related to the defects of the energy bands, which can be seen in the form of an effective mass change of the electron (or the charge carrier).

Furthermore, the real part of the relative permittivity (ϵ_r_ = *n*^2^ + *K*^2^; *n* is the refractive index and *K* is the extinction coefficient), and its imaginary part (ϵ_i_ = 2*nK*) of the E478 epoxy resin are obtained after measuring *K* and *n* from the equations:^27^
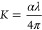
9
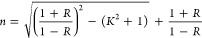
10In this study, a JASCO V-770 double-beam UV/VIS/NIR Spectrophotometer from JASCO Corporation (Tokyo, Japan) was used to obtain the *T*(λ)_%_ and *R*(λ)_%_ values. The instrument is supplied with a deuterium and wolfram-halogen lamp, and the tested wavelength range was 200–800 nm.

## Results and Discussion

### Elemental Analysis

**Figure 5:**
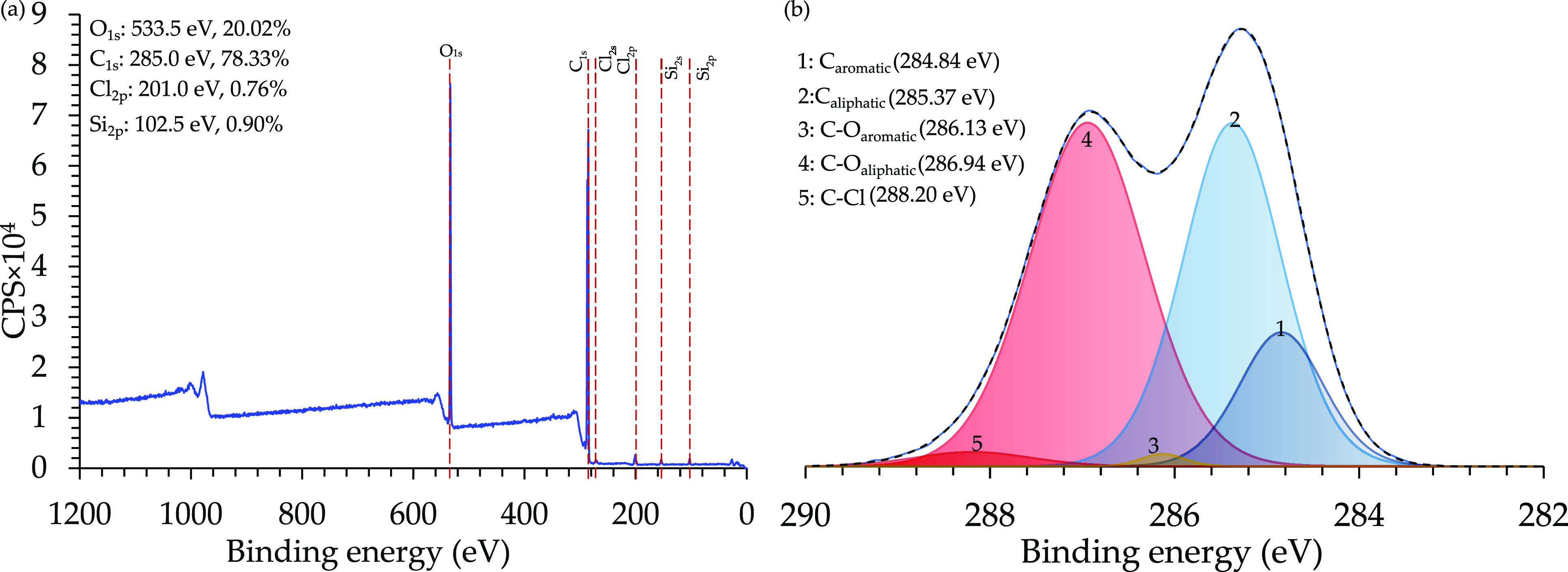
XPS (a) wide spectrum, and (b) C_1*s*_ high-resolution spectrum for the E478 single-component epoxy resin.

The XPS wide spectrum is presented in Figure 5a. The spectrum was calibrated to a carbon peak at a binding energy of 285.0 eV, consistent with the literature about epoxy resins.^28−30^ The results show the existence of oxygen (20.02%) and carbon (78.33%) as the main components of the epoxy resin structure. Moreover, the existence of chlorine (0.76%) as the termination agent,^31,32^ and silicon (0.90%) as the networking agent.^33,34^ It is important to mention the absence of copper in the spectrum (Cu_2p3_ is located at a binding energy of 932.7 eV^35^), reporting pure photoemission of electrons from the scanned regions of interest.

The C_1s_ high-resolution spectrum is presented in Figure 5(b). The fitted peaks show the existence of the aromatic C=C bonds, which have delocalized electrons within the benzene ring structure.^36^ The aromatic rings play a significant role in the field emission of electrons from metallic-organic composite field emitters. In principle, the coating layer acts as an additional potential energy barrier at the emitter apex surface when its thickness is in the range of a few nanometers, such as in the case of metallic-oxide composite emitters.

Moreover, when the coating layer is composed of a polymer material of a thickness of tens of nanometers, the electrons classically tunnel through the polymer layer (epoxy resin in our study). According to Knápek *et al*.,^23^ this is possible because of the creation of an electron vent (conduction channel) connecting the metallic surface to the vacuum. The intense electrostatic field passes through the epoxy layer, reorients the electric dipoles, merges the oriented electric dipoles, forms nanocapacitors, and finally the nanocapacitors merge forming the electron vent. However, the nature and formation of these nanocapacitors was not discussed in Knápek’s work.

In our theory, the existence of the aromatic rings including the delocalized electrons within the epoxy structure is the source of these nanocapacitors. From this point, the nanocapacitors are referred to as molecular capacitors. Each aromatic ring stores three π-delocalized electrons alternating between the six carbon atoms, forming a molecular capacitor. Under the influence of an external electrostatic field, which has the highest magnitude at the apex of the emitter, the chemical bonds within the cured epoxy structure are stretched and have different lengths, frequencies, and electronic features.^37^ This allows a possible connection between the aromatic rings (or even possible destruction of the ring bonds), forming larger molecular capacitors, and a higher probability for the stored electrons to alternate between the joint rings through a possible broken C–H or C–O bond. This assumption explains the switch-on phenomenon, thermionic energy distribution, and the focused electron beam of CFE from metallic-epoxy composites.^4,23^

**Figure 6:**
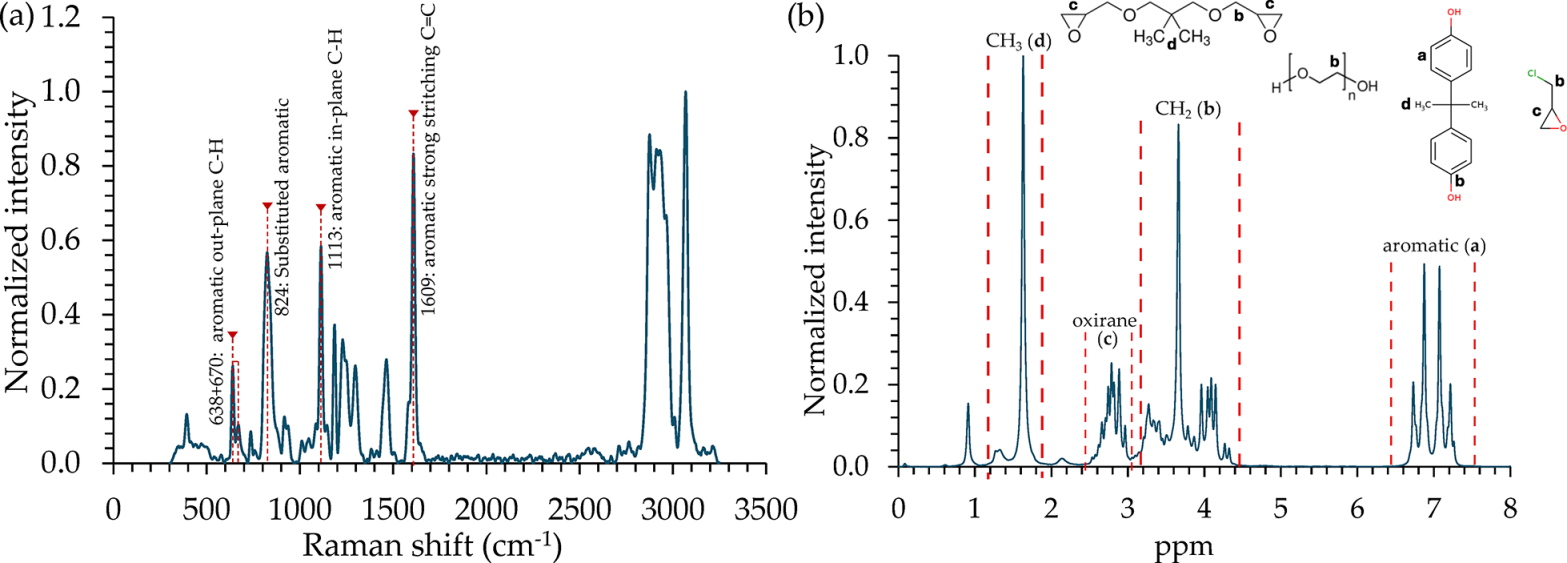
Structural analysis of the E478 single-component epoxy resin presented by (a) Raman spectroscopy and (b) ^1^H-NMR spectroscopy.

Supporting the XPS analysis, the normalized Raman spectrum was measured and is presented in Figure 6(a). The results are equivalent to previously reported spectra for DGEBA epoxy resin precursor after curing.^38,39^ The most important peaks for this study are the peaks related to the aromatic rings located at 638 and 670 cm^–1^ for the aromatic out-plane C–H bond, 824 cm^–1^ for the aromatic substitutions, 1113 cm^–1^ for an aromatic in-plane C–H bond, and 1609 cm^–1^ for the aromatic strong stretching of the C=C bond. The intensity and area of the highlighted peaks show the major content of the aromatic rings within the epoxy structure.

These results support the XPS analysis and the assumption that these aromatic rings are the source of the generated molecular capacitors and electrons vent. For more discussion about the other peaks check.^38^ Moreover, the 1H-NMR spectrum is presented in Figure 6(b). The aromatic rings are found clearly at 7 ± 0.2 ppm, and according to the SDS, the source of the aromatic rings is bisphenol A. The peaks at 3.2–4.4 ppm are related to the CH_2_ bonds in the neopentyl glycol diglycidyl ether, polyglycol, and epichlorohydrin. The peaks at 2.4–3.0 ppm are related to the oxirane groups in the neopentyl glycol diglycidyl ether and the epichlorohydrin. Finally, the peak at 1.6 ppm is related to the CH_3_ in the Bisphenol A. This shows high consistency with the XPS and Raman results about the existence and the source of the aromatic rings, which is mainly from the cured epoxy resin structure.^40−44^

These results are important to understand the chemical resistance of the E478 single-component epoxy resin for possible future applications in electrochemical scanning tunneling microscopy (EC-STM) to study the chemical reactions at the metal-liquid interface. The planned EC-STM will be prepared from tungsten-E478 composite nanotips with a controlled clean apex size.

### UPS Analysis

The obtained results from the UPS analysis are important to understand the charge carrier impregnation from the metallic surface to the resin coating layer of a composite field emitter. Moreover, it is essential to obtain accurate CFE analysis results.

**Figure 7:**
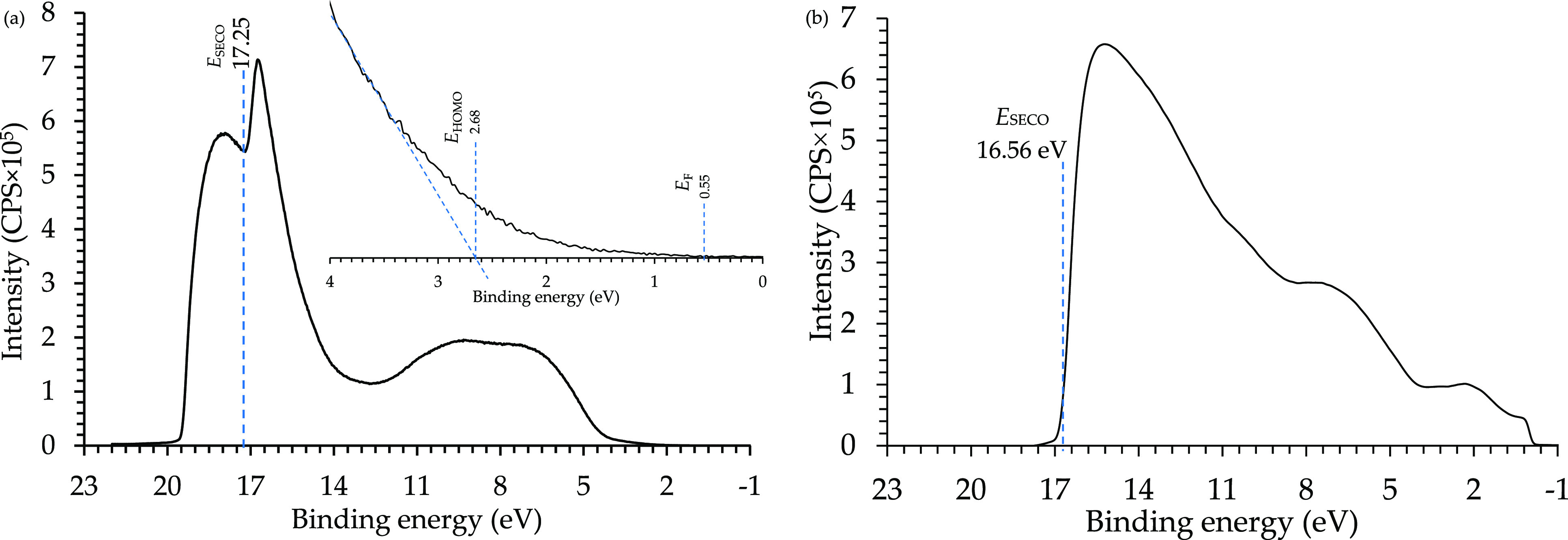
UPS analyses for the (a) E478 epoxy resin and (b) tungsten foil.

According to the E478 epoxy resin UPS spectrum in Figure 7(a), *E*_SECO_ = 17.25 eV, *E*_F_ = 0.55 eV, and *E*_HOMO_ = 2.68 eV. Substituting these into eqs 1 and 2 provides ϕ_E478_ = 3.42 eV and ψ_E478_ = 6.1 eV. Moreover, the tungsten (W) UPS spectrum is presented in Figure 7(b), where *E*_SECO_ = 16.56 eV and *E*_F_ = 0 eV, providing ϕ_W_ = 4.66 eV. Aligning the Fermi level of both materials provides a difference in the vacuum level of 1.24 eV. To complete the energy diagram for the E478 epoxy resin and tungsten-E478 interface, UV/VIS spectroscopy was followed to measure the energy gap and its characteristics, which was then combined with the UPS analysis to study the possible excitation energies.

### UV/VIS Analysis

**Figure 8:**
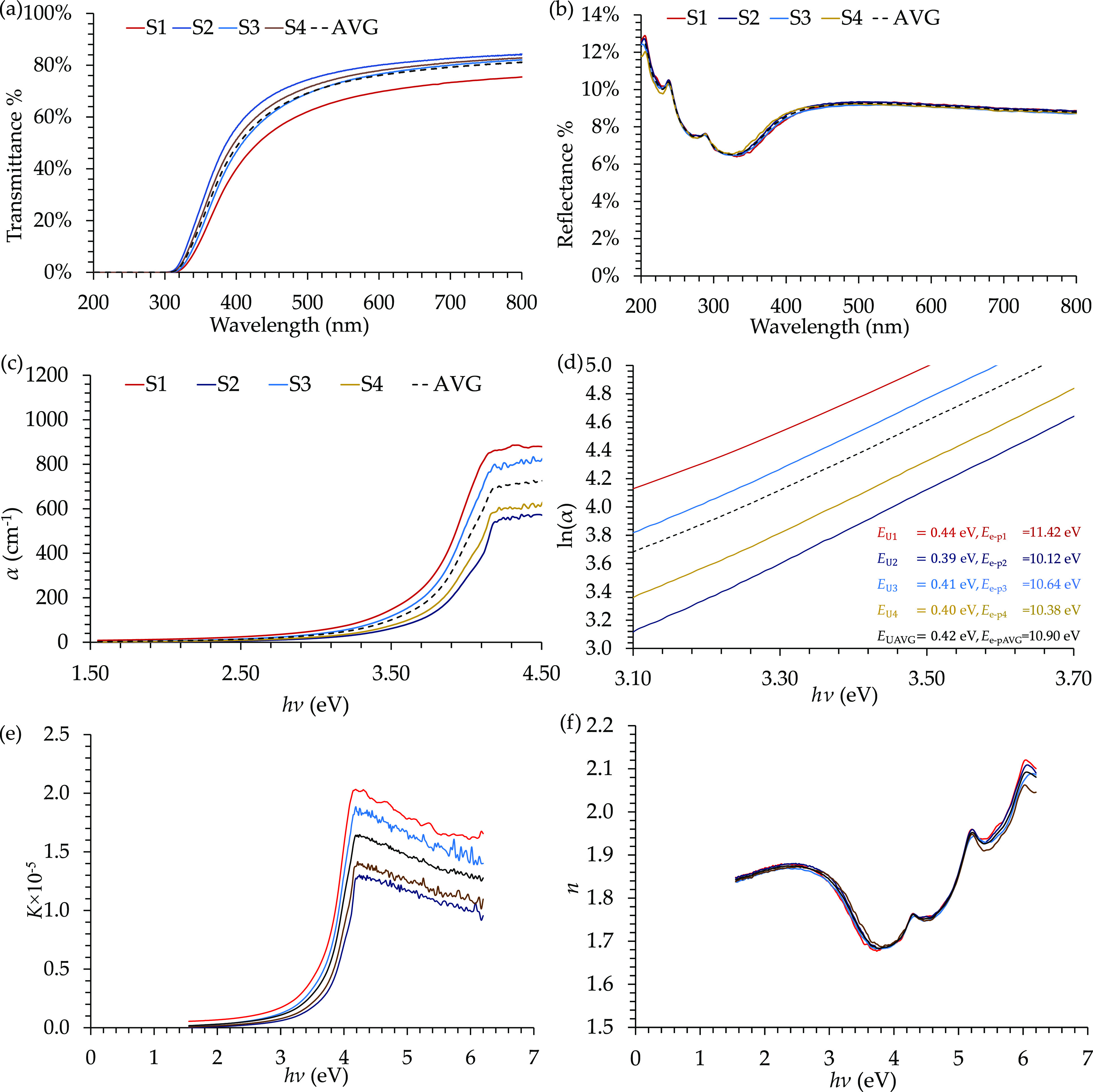
UV/VIS analyses for the E478 epoxy resin. Presented results include (a) transmittance, (b) reflectance, (c) absorption coefficient, (d) Urbach plot, (e) extinction coefficient, and (f) refractive index.

**Figure 9:**
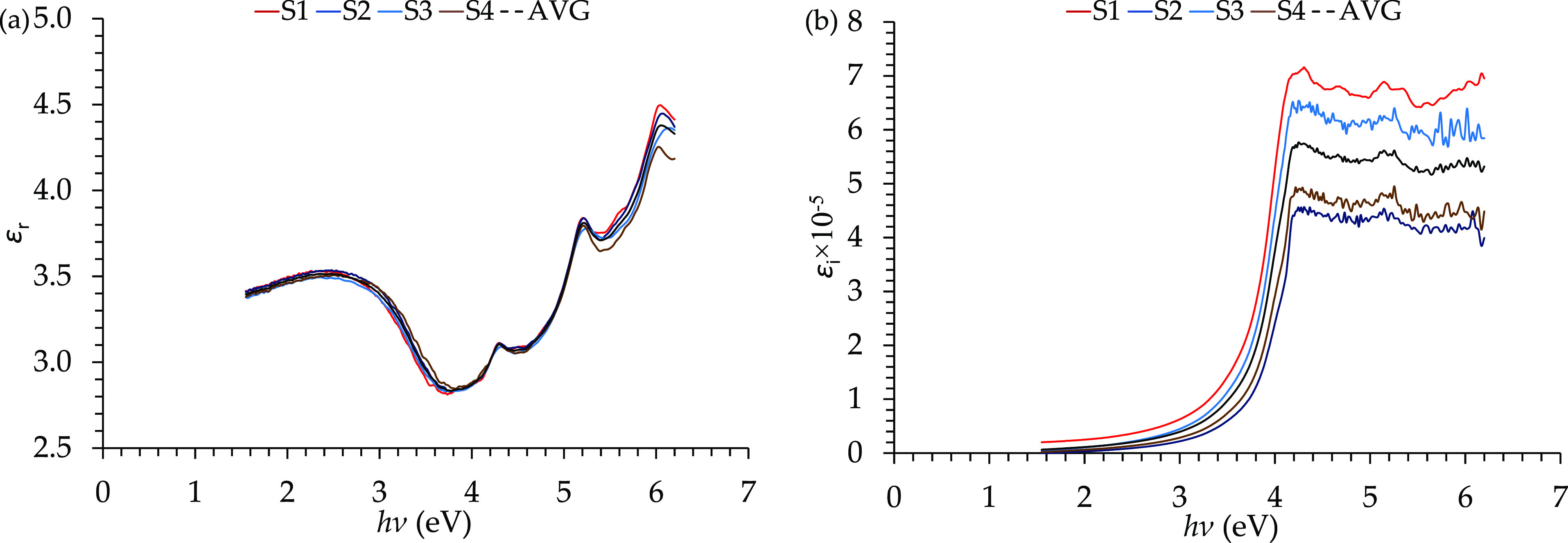
Relative permittivity of the E478 epoxy resin presented by its (a) real part and (b) imaginary part.

The optical characteristics of the E478 epoxy resin are presented in Figure 8. *T* and *R*(Figure 8(a and b)) were substituted into eq 3 to obtain the function α(λ), which is presented in Figure 8(c). Applying the natural logarithm to α, the linear part of the ln(α) vs *hν* curve was detected at the near UV region of photon energy 3.1–3.7 eV, as presented in Figure 8(d). Analyzing the linear parts for the four films provides Urbach tailing energies of *E*_U_ ≈ 0.44, 0.39, 0.41, and 0.40 eV, with an average value of 0.42 eV. Moreover, the electron–phonon interaction had the values of *E*_*e*–*p*_ ≈ 11.42, 10.12, 10.64, and 10.38 eV, with an average value of 10.90 eV. The extinction coefficient and the refractive index of the E478 epoxy resin were then measured from α and *R*, and the results are presented in Figure 8(e and f). *K* and *n* values were then used to obtain the real and imaginary parts of the relative permittivity (Figure 9(a and b)). Three peaks were detected at the following (*hν*, ϵ_r_) coordinate points: (4.34 eV, 3.1), (5.19 eV, 3.8), and (6.02 eV, 4.4).

**Figure 10:**
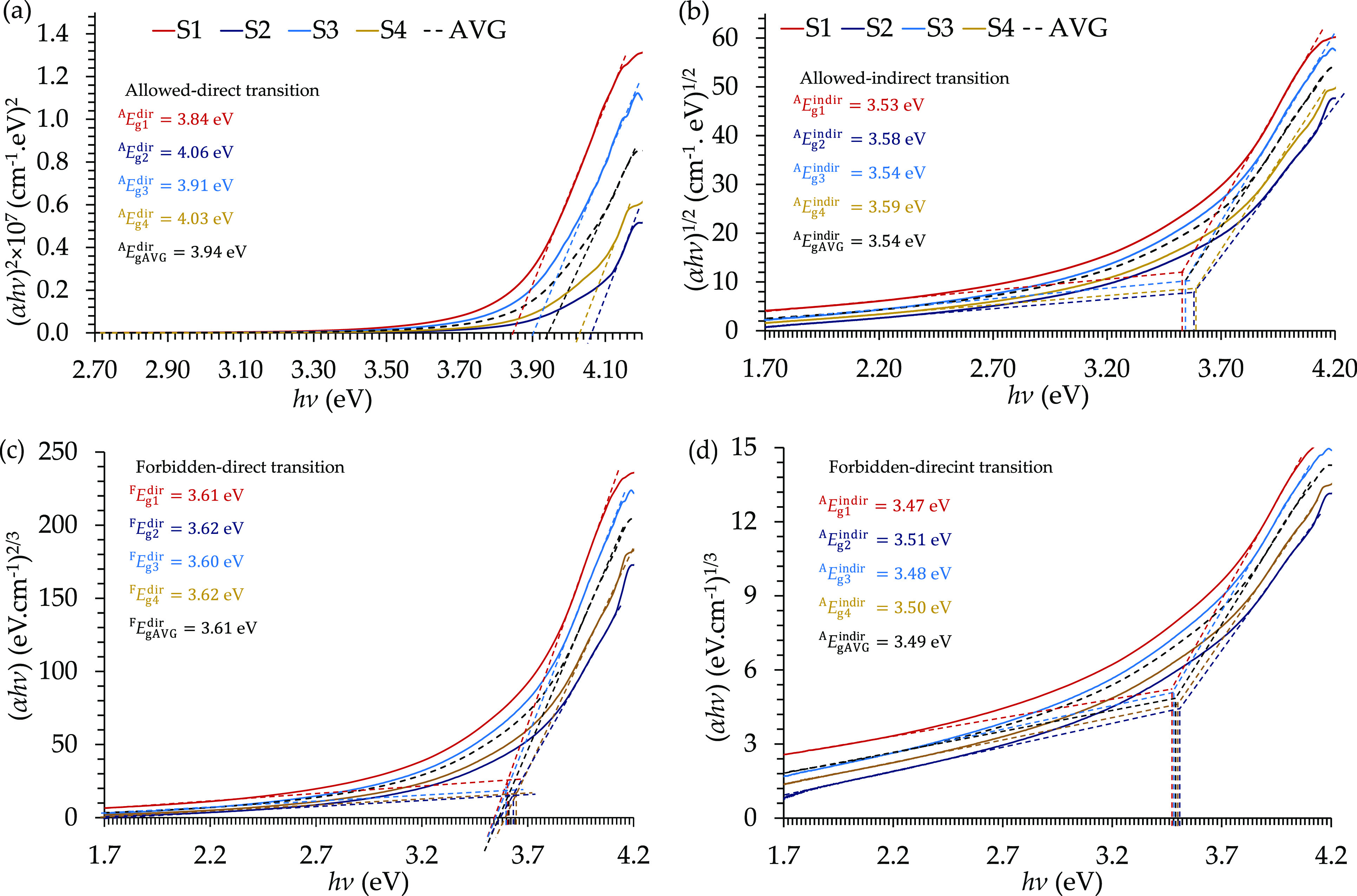
Allowed direct and indirect electron transition in the E478 epoxy resin presented by the (a) allowed direct Tauc plot, (b) allowed indirect Tauc plot, (c) allowed direct and indirect transitions in the momentum-space energy diagram, and (d) Urbach tailing in the density of states-energy diagram.

Furthermore, the Tauc plots of the allowed/forbidden direct/indirect transitions are presented in Figure 10. The allowed direct energy gap values for the four films were measured from the curves in Figure 10(a) and had values of ^A^*E*_g_^dir^ = 3.84, 4.06, 3.91, and 4.03 eV, with an average value of 3.94 eV. Moreover, the allowed indirect energy gap values were measured from the curves in Figure 10(b) and had values of ^A^*E*_g_^indir^ = 3.53, 3.58, 3.54, and 3.59 eV, with an average value of 3.54 eV.

Besides, the forbidden direct energy gap values were reported from the curves in Figure 10(c) and had the values of ^F^*E*_g_^dir^ = 3.61, 3.62, 3.60, and 3.62 eV, with an average value of 3.61 eV. Furthermore, the forbidden indirect energy gap values (Figure 10(d)) reported the values of ^F^*E*_g_^indir^ = 3.47, 3.51, 3.48, and 3.50 eV, with an average value of 3.49 eV.

Combining the results obtained from UPS and UV/VIS analyses, the lowest unoccupied molecular orbital *E*_LUMO_ (or the bottom of the conduction band) was measured 1.26 eV above the Fermi level of the E478 epoxy resin. Thus, the electron affinity χ was reported to be 2.16 eV. A summary of the obtained excitation energies from UPS and UV/VIS is presented in Table 1.

**Table 1:** Summary of the Excitation Energies As Obtained from the UPS (Figure7) and UV/VIS (Figure 10) Analyses of the E478 Epoxy Resin^a^

ϕ	ψ	^A^*E*_g_^dir^	^A^*E*_g_^indir^	^F^*E*_g_^dir^	^F^*E*_g_^indir^	*E*_HOMO_	*E*_LUMO_	χ
3.42	6.10	3.94	3.61	3.54	3.49	–2.68	+1.26	2.16

a All energies are measured in eV.

**Figure 11:**
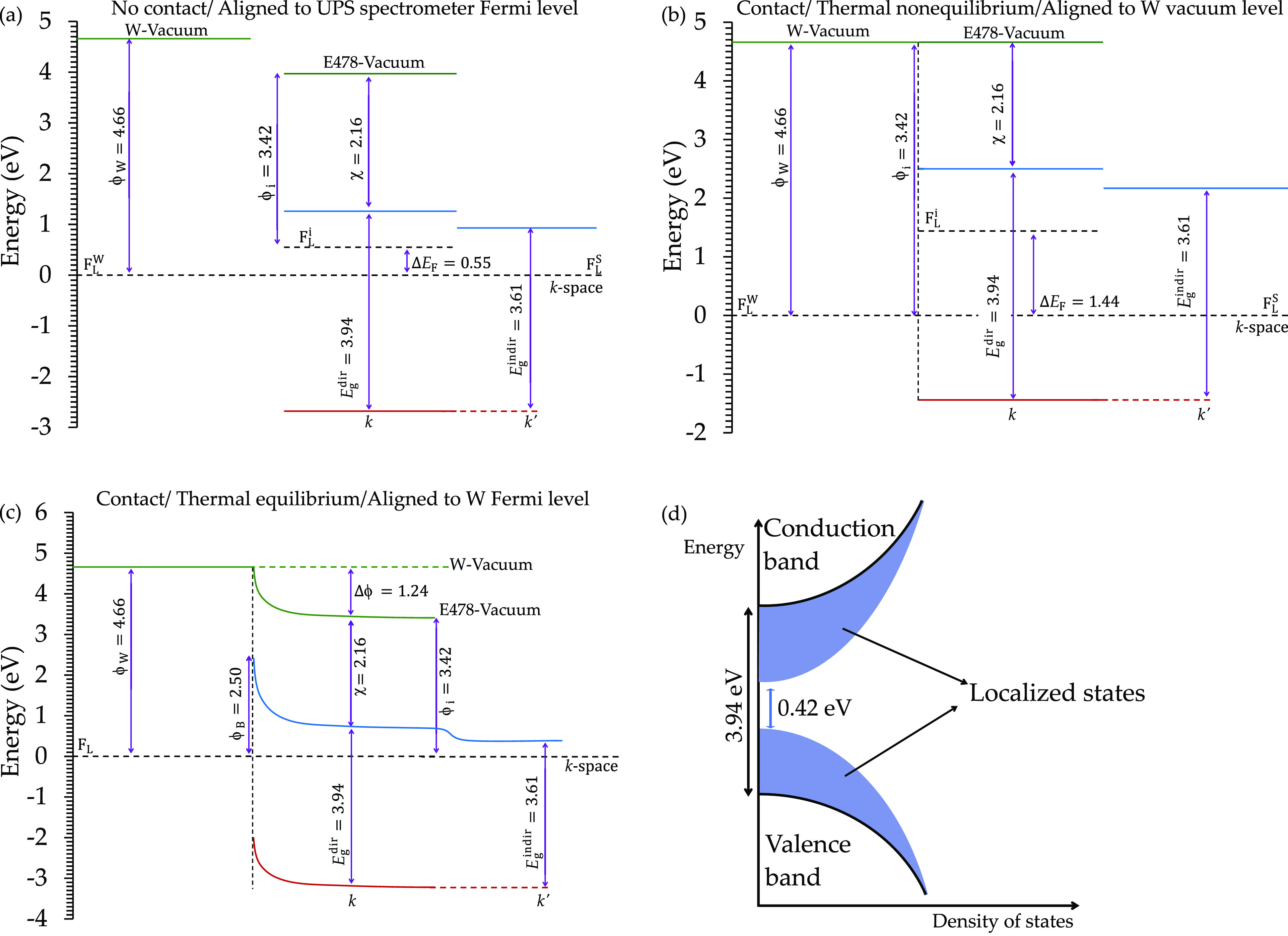
Energy diagram for a tungsten-E478 epoxy resin composite electron source shows the energy band alignment when the tungsten and the epoxy resin are (a) not in contact, (b) at thermal nonequilibrium contact, and (c) at thermal equilibrium contact. Moreover, (d) the Urbach tailing for the epoxy resin shows defects in the energy bands due to the amorphous structure and the impurities in the epoxy resin.

On top of that, combining the data presented in Table 1 and the UPS results of the tungsten provides the energy band diagram for the tungsten-E478 epoxy resin composite electron source. The epoxy resin was considered an n-type semiconductor because its Fermi level is closer to that of the LUMO. Before the tungsten and the epoxy were in contact, the energies were aligned to the UPS spectrometer Fermi level, and the energy band diagram of this case is presented in Figure 11(a). In this case, the Fermi level difference between tungsten and the epoxy resin was 0.55 eV.

After the sample was in thermal nonequilibrium contact, the energies were aligned to the tungsten vacuum level. The results are presented in Figure 11(b), and the difference in Fermi levels was increased to 1.44 eV. Moreover, after being in thermal equilibrium contact (Figure 11(c)), the Fermi levels were aligned to the tungsten Fermi level, reporting a Schottky barrier of height ϕ_B_ = 2.50 eV, and a difference in vacuum levels of 1.24 eV. Moreover, the Urbach tailing energy at a very low density of states indicates a large reduction in the energy gap value (3.94–0.42 eV) because of the defects and delocalized energy levels of the LUMO and HOMO (check Figure 11(d)). This is an important point because during the emission process electrons are impregnated inside the epoxy structure from tungsten. The impregnated electrons are then stored inside the formed molecular capacitors (as discussed in the XPS results).

**Figure 12:**
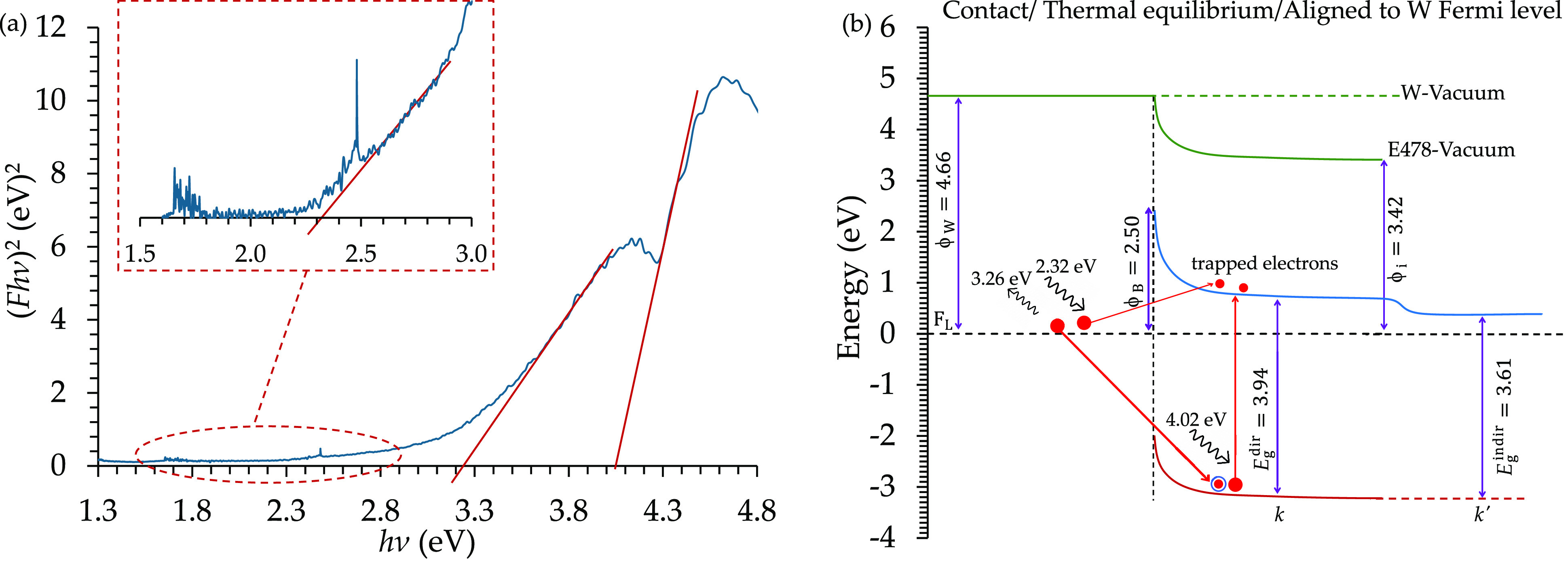
UV/VIS analysis for a tungsten foil coated with E478 epoxy resin.

The results were approved by coating a tungsten foil with an epoxy resin. The layer thickness was 0.095 mm, and the Kubelka–Munk function *F* = (1 – *R*)^2^/2*R* was used to obtain the related UV/VIS Tauc plot ((F*h*ν)^2^ vs *hν* in this case). The obtained Tauc plot is presented in Figure 12(a), showing three edges corresponding to energies of 2.32, 3.26, and 4.04 eV. Comparing the obtained edges with the energy diagram in Figure 11(c) shows that the edge of: 2.32 eV is related to an excited electron from the tungsten Fermi level to the epoxy LUMO, and 4.02 eV is for an electron excited from the epoxy HOMO to its LUMO. However, the 3.26 eV photon energy was for a photon emitted from an electron that occupied the free hole in the epoxy HOMO from the tungsten Fermi level, trapping electrons in the epoxy conduction band. The three possible electron transitions are listed in Figure 12(b).

This helps to increase the electron’s occupation within the epoxy structure, reducing the density of unoccupied energy states and the energy gap. When the density of states is low enough, the switch-on phenomenon occurs, causing a sudden high emitted electrical current density. Furthermore, The enhancement in the external electrostatic field has its maximum at the apex of the emitter. Thus, the generated electron vent forms a conductive microchannel that allows the electrons to “classically/thermally” tunnel through the insulating layer to the vacuum. Besides, the electron vent is conductive, which caused the loss of the insulating characteristics of this part of epoxy resin gaining conductive characteristics that form an additional potential energy barrier at the vent crater, which works as an induced convergence electrical lens. This forms a triple regime field emission process that includes cold field emission at the tungsten-epoxy interface (impregnation of electrons from tungsten to epoxy), thermal transport of the electrons through the epoxy layer, and thermally assisted field emission of a condensed electron beam at the crater-vacuum interface.

## Conclusion

This study provides a comprehensive analysis of the E478 single-component epoxy resin. The XPS provided a brief elemental analysis for the studied epoxy, showing that it is mainly composed of carbon, oxygen, chlorine, and silicon. The carbon high-resolution peak showed a high contribution of the organic aromatic rings in the epoxy structure, which was connected to the creation of the molecular capacitors when the epoxy is subjected to an intense external electrostatic field. Moreover, the XPS results were supported by Raman and hydrogen nuclear magnetic resonance spectroscopy, showing the major contribution of the aromatic rings in the cured epoxy structure.

The UV/VIS analysis provided optical and energy gap characteristics. The results showed that the E478 epoxy resin is considered a high-order semiconductor with an average energy gap of 3.95 eV, Urbach tailing of 0.42 eV, and electron–phonon interaction of 10.9 eV. The UPS analysis reported a local work function of 3.42 eV and an ionization potential of 6.1 eV. Moreover, UV/VIS analysis was combined with the UPS analysis to report the electron affinity of the E478 epoxy resin, to study its energy band diagram, and the energy band diagram of the tungsten-epoxy interface.

The results of this study are important to understand the cold field emission regime of composite metallic-(organic insulator) electron sources (tungsten-epoxy in this study). Moreover, the elemental and structural analyses are important to understand the high chemical resistance of the E478 single-component epoxy resin, which is important in several applications. The results will be used for future studies to study the cold field emission characteristics from tungsten-E478 electron sources, with possible applications in X-ray generation from liquid metals for computed tomography, electrochemical scanning tunneling microscopy, biosensing applications, and the generation of focused and stable electron beams for linear accelerators and electron beam therapy.
